# Peripartum cardiomyopathy: Clinical Predictors of favourable maternal-fetal outcomes

**DOI:** 10.12669/pjms.42.5.13114

**Published:** 2026-05

**Authors:** Zahoor Ahmad Khan, Farnaz, Saira Jabeen

**Affiliations:** 1 Zahoor Ahmad Khan, FCPS(Cardiology), FCPS (Clinical Cardiac Electrophysiology).Associate Professor, Department of Cardiac Electrophysiology,MTI, Hayatabad Medical Complex, Peshawar, Pakistan; 2 Farnaz, FCPS.Associate Professor, Department of OBGYN, Lady Reading Hospital MTI Peshawar,Peshawar, Pakistan; 3 Saira Jabeen,Resident OBGYN, Department of OBGYN, Lady Reading Hospital MTI Peshawar,Peshawar, Pakistan

**Keywords:** Atrial fibrillation, Ejection fraction, Pulmonary hypertension, Peripartum cardiomyopathy

## Abstract

**Objectives:**

To evaluate maternal cardiac complications fetal outcomes, and delivery considerations in patients diagnosed with peripartum cardiomyopathy(PPCM) in tertiary hospital.

**Methodology:**

It was a prospective observational study, conducted in the Department of Obstetrics and Gynecology, Lady Reading Hospital, Peshawar, from January 17, 2024 to December 17, 2024 after obtaining ethical approval. It included all patients with PPCM admitted via emergency or OPD. Peripartum cardiomyopathy was diagnosed according to European Society of Cardiology criteria, defined as heart failure occurring toward the end of pregnancy or in the months following delivery, with left ventricular ejection fraction <45%, in the absence of another identifiable cause of cardiomyopathy. A total of 46 patients were enrolled. Patients with pre-existing dilated cardiomyopathy, chronic obstructive pulmonary disease and significant congenital valvular heart disease had been excluded. All patients were managed by a Multidisciplinary team, including a consultant Obstetrician, Consultant Cardiologist, a Cardiac Electrophysiologist, an anaesthetist, an intensivist and a paediatrician in a tertiary hospital. Primary outcome were Maternal cardiac complications and mortality. While secondary outcomes were Fetal outcomes (live birth, preterm birth, IUFD, miscarriage) and mode of delivery. Predictors and outcomes of PPCM in patients were assessed, i.e. ejection fraction, fractional shortening, BMI, pre-eclampsia, multiple pregnancy, parity and age. We categorized our sample into two groups based on presence or absence of defined outcomes of cardiac complications and compare predictors between these two groups.

**Results:**

Clinical characteristics observed in patients with relatively stable outcomes included normal BMI, mean age less than 30 years, mean ejection fraction of 37% with no thrombus, no pre-eclampsia or multiple pregnancy. The mode of delivery preferred was elective c-section 65.2% (n=30) decided by MDT (cardiologist, anaesthetist, and obstetrician) to avoid stress of bearing down during labor if EF less than 37.7%. The most common cardiac complication was pulmonary hypertension. The Neonatal outcomes included 71.8% live births, IUFD 19.6% and miscarriages 8.7%.

**Conclusion:**

Maternal outcomes were generally favorable when patients with peripartum cardiomyopathy were managed at a tertiary care center through a multidisciplinary team approach. Better outcomes were commonly observed in patients with normal BMI, mean left ventricular ejection fraction ≥37.7%, absence of pre-eclapmia and multiple pregnancy.

## INTRODUCTION

PPCM is the most common cardiomyopathy in pregnancy.^1^ PPCM is defined by the European Society of Cardiology (ESC) as an idiopathic cardiomyopathy presenting with Heart Failure (HF) secondary to left ventricular (LV) systolic dysfunction towards the end of pregnancy or in the months following delivery, where no other cause of HF is found. It is a diagnosis of exclusion; the ejection fraction (EF) is nearly always reduced below 45%.”.^2^,^3^ Globally, the Frequency of cardiac disease in pregnant women is 16.7%^2^ with peripartum cardiomyopathy 4.1%.^3^

Mortality in PPCM varies from <2% to 50%. Ventricular arrhythmias, heart failure, stroke and death are some of complications found in 39%-60% of PPCM patients who have additional underlyng risk factors like eclampsia or gestational hypertension.^4^ Common presenting symptoms vary from shortness of breath, fatigue, palpitations, chest pain, and fluid retention^5^,^6^ to severe heart failure or life-threatening arrhythmias. Managing cardiac diseases in pregnancy requires a multidisciplinary approach involving obstetricians, cardiologists, anesthesiologists, and neonatologists. Treatment strategies aim to optimise maternal health while ensuring the well-being of the fetus.^7^

In some cases, medication adjustments are necessary to manage hypertension, arrhythmias, or heart failure and balance the benefits of medication with potential risks to the fetus.^8^ Regular cardiac monitoring through echocardiography and electrocardiography is crucial to assess maternal and fetal well-being. There is no such literature available on this subject at our regional level. The goal of this study is to determine the frequency and outcomes of peripartum cardiomyopathy. The results of this study will help understand these cardiac diseases in pregnancy and provide timely, specialized care to improve the chances of a safe and successful pregnancy for women with heart conditions.

## METHODOLOGY

We conducted this study in the department of obstetrics & Gynaecology, Lady Reading Hospital, Peshawar, from January 17, 2024 to December 17, 2024.

### Ethical Approval:

It got approval from the Institutional Ethical Board reference no 1005/MTI/LRH; dated December 27, 2023.

### Inclusion & Exclusion Criteria:

We included all patients presenting with PPCM in six months’ time, admitted via emergency triage or OPD at Lady Reading Hospital. A total of 46 patients were included in study. Patients with pre-existing dilated cardiomyopathy, chronic obstructive pulmonary disease and significant congenital valvular heart disease have been excluded.

Primary outcome were Maternal cardiac complications and mortality. While secondary outcomes were Fetal outcomes (live birth, preterm birth, IUFD, miscarriage) and mode of delivery. Maternal cardiac complications included: Acute heart failure, Pulmonary edema, Pulmonary hypertension, arrhythmias, and maternal mortality. Patients who remained hemodynamically stable and did not develop the above complications, no requirement for mechanical ventilation or prolonged ICU admission and no maternal mortality were considered to have favourable outcomes, indicating control of PPCM-related cardiac complications.

Informed consent was obtained from all women, explaining to them the objective of this study, ensuring their confidentiality of the information and that there is no risk involved to the patient while participating in this prospective observational study.

Demographic detail like age, parity, gestational age at time of presentation of symptoms/signs of PPCM, BMI, address, education status, were recorded, comorbid were noted down. Echocardiography was done, and the Ejection fraction was noted. Pregnant patients diagnosed with PPCM were included in the study and were admitted to the High Dependency Unit Obstetric department, for workup and planning mode and time of delivery. An experienced consultant with a minimum of five years of post-fellowship experience supervised the whole process in liaison with cardiologist and anaesthetist. Each patient’s details were recorded on an approved proforma.

The analysis was performed by using SPSS v.21 software. Mean ± SD were presented for numerical variables such as age, weight, height, and BMI. Frequencies and percentages were presented for categorical variables such as education status, type of admission, ICU admission, mode of delivery, maternal cardiac complication and fetal outcomes. Post-stratification chi-square test was conducted at 5% level of significance. Results obtained from this study were shown in the form of tables. We categorized our sample into two groups based on presence or absence of defined outcomes of cardiac complications and compare predictors between these two groups.

## RESULTS

The data were analyzed after entering the completed questionnaire in SPSS. Descriptive inferential statistic were computed on the basis of demographic sheet (N=46) are given in Table-I and Table-II. The mean ejection fraction of these patients was 37.70 % ±6.998SD and the most common mode of delivery was elective cesarean section in 69.6% (n=32) under general anaesthesia and of these, 28.3% were intubated and shifted to the ICU. Table-III shows the cardiac complications in which Pulmonary hypertensin was highest 37%(n=17) followed by acute cardiac failure, pulmonary edema and atrial fibrillation. Table-IV: PPCM risk factors comparison with/without Cardiac Complications showed that Cardiac complication.in all pre-eclampsia patients 6 (18.2%) and patient with less than 34.7%EF. Table -V(a) and fetal outcomes in Table-V(b) shows more IUFD and miscarriages in cardiac complication group.

**Table-I:** Descriptive statistics (n = 46). Mean, Standard Deviation.

Variables	Mean	Std. Deviation
Age (Years)	28.52	6.214
BMI (Kg/m^2^)	24.9707	1.32492
Parity	3.67	1.86
Ejection fraction%	37.70	6.998
Length of stay in hospital days	5.45	2.956

**Table-II:** Descriptive statistics (n = 46) frequency and percentage.

Categorical variable (n=46)	Frequency	Percentage
Booked	1	2.2%
Unbooked	45	97.8%
Emergency admission	41	89.1%
Elective admission	5	10.9%
***Any prior cardiologist consultation about her disease***		
No	15	32.6%
Yes	31	67.4%
Mode of delivery NVD	12	26.1
Cesarean section	32	69.6%
***Presentation in trimester***		
First-trimester presentation	2	4.3%
Second-trimester presentation	2	4.3%
Third-trimester presentation	42	91.3%
Maternal ICU admission	13	28.3%
Maternal PPH	10	21.7%

**Table-III:** Maternal Cardiac Complications.

Maternal Cardiac Complication	Frequency	Percentage
Acute Cardiac Failure	4	8.4%
Pulmonary edema	4	8.7%
Pulmonary hypertension	17	37%
Arrhythmia: Atrioventricular Nodal re-entrant tachycardia (AVNRT)	1	2.2%
Arrhythmia: Paroxysmal Atrial Fibrillation (AF)	2	4.3%
Arrythmia: Ventricular Tachycardia (VT)	4	8.7%
Maternal death	1	2.2%
No complications	13	28.3%

**Table-IV:** PPCM risk factors with/without Cardiac Complications

	Maternal Complications (Yes)	Maternal Complications (n=No)	P value
Admission type			0.095
Elective admission for delivery	2 (6.1%)	3 (23.1%)	
Emergency admission for delivery	31 (93.9%)	10 (76.9%)	
Booking status			
Booked	1 (3.0%)	0	0.526
Unbooked	32 (71.1%)	13 (28.9%)	
Maternal ICU admission			0.008
Yes	13 (39.40%)	0	
No	20 (60.60%)	13 (39.4%)	
Mode of Delivery			0.004
NVD	5 (15.2%)	8 (61.5%)	
Caesarean Section	27 (81.8%)	4 (30.8%)	
Miscarriage	1 (3.0%)	1 (7.7%)	
Pre-eclampsia	6 (18.2%)	0	0.09
Multi-pregnancy	7(21.2%)	3(23.1%)	0.89
Ejection fraction			0.005
Less than 34.7%	14 (100%)	0	
More than 34.7%	19 (59.4%)	13(40.6%)	

**Table-V(a):** Fetal outcomes in peripartum cardiomyopathy.

Fetal outcomes	Frequency	Percentage
Term	21	45.7%
Preterm	12	26.1%
IUFD	9	19.6%
Miscarriage	4	8.7%

**Table-V(b):** Fetal outcomes in peripartum cardiomyopathy with and without cardiac complications.

Fetal Outcomes	PPCM with complications Yes	PPCM with complications No	P-Value
Term	15 (71.40%)	6 (28.60%)	0.008
Preterm	09 (75.00%)	3 (25.00%)	
IUFD	05 (56.60%)	4 (44.40%)	
Miscarriage	04 (100%)	0	

A *t-test* for independent sample (Levene’s Test for Equality of Variance can be assumed p<0.00) for ejection fraction comparison with two group of PPCM with /without cardiac complication was calculated and showed statistically significant difference of mean exist between cardiac versus non cardiac complication t= -6.328 p= 0.000, 95% Confidence interval [-13.990, -7.231]. Maternal cardiac complication were observed when EF was less than 34.7% versus no complication when >45.31%.

## DISCUSSION

A total of 38,820 deliveries were conducted during the study period in the OBGYN department. PPCM was confirmed in 46 cases with an incidence of 1.18 per 1000 deliveries. Globally, PPCM is reported to be approximately one per 15,000 live births in Japan and one per 1000 live births in South Africa,^9^ which is the same as our study. Asian populations PPCM range from approximately 1 in 837 deliveries in Pakistan to 1 in 20,000 deliveries in Japan.^10^,^11^

Various risk factors have been described in the PPCM. Women at increased risk include those with African ancestry, primiparous mothers younger than 18 years, advanced maternal age, multiparity, twin pregnancy and preeclampsia.^12^ The mean age of presentation in this study was 28 years, although it can occur at any age .Advanced maternal age more than 30 years is an important risk factor for PPCM.^11^ One study found the mean age to be 33 years and predicted unfavorable outcomes.^11^ A study by Kolte et al. showed a 10-fold increased risk in females over 40 years.^12^ Parity of four or more than four is considered a risk factor for PPCM in different studies^11^-^13^ and in this study, mean parity was 3.67, pre-eclampsia was seen in 18.2% (n=6)and multiple pregnancy in 21.7%(n=10) with PPCM.. According to a meta-analysis conducted, the prevalence of PE was 22% among patients with PPCM and three times higher than the global prevalence of pre-eclampsia, which is estimated at 7.5%.^14^

In this study, cardiac complication was seen in all patients when ejection fraction was less than 34.70% and no complication ≥ 45.31%. When ejection fraction was more than 35 % complication seen were mostly in patient who had risk factor of pre-eclampsia and multiple pregnancy. In another study when the ejection fraction was ≤ 30 %, mortality was 7% to 15% of cases globally 14-18. Our study cardiac complications of pulmonary hypertension 37% (n=17), cardiac failure 10.9% (n=5), and arrhythmia which were managed by MDT and survived. Only one mortality was seen due to complication of pre-eclampsia and multiple organ failure.^19^

Mode of delivery also impacts the cardiac outcomes. Deciding mode, depends on hemo-dynamic stability, so early delivery or pregnancy termination had to be performed in case of unstable patients. In this study, pregnancy was interrupted before term due to cardiac instability in 26.1% (n=12) and pregnancy was terminated in first trimester in 8.7% (n=4). Vaginal delivery is preferred in case of hemo-dynamic stability and F >45%, provided epidural anaesthesia is administered to avoid sudden blood pressure increase 17. The decision E regarding the mode of delivery was made by a multidisciplinary team based on maternal hemodynamic stability, obstetric indications, and fetal status. Emergency caesarean sections were primarily performed in patients with maternal cardiac instability presented in labor. In this study, 69.6% (n=32) underwent emergency Caesarean section due to presentation in active labor and hemo-dynamically unstable in laison with MDT team of cardiologist, obstetrician and anaesthetist. Of these sections, 28.3% (n=13) were shifted to the ICU and later improved and extubated to shift back to the ward. Only one patient died in the ICU after 48 hrs from multiorgan failure.

The global prevalence of arrhythmia in PPCM is 19%, seen in a study on 9,841 PPCM, where ventricular arrhythmia were the most common ones (4% for VT, 1% for VF), followed by supraventricular arrhythmia (1.3% for AF, 0.5% each for atrial flutter and atrial tachycardia).^20^,^21^ In this study, arrhythmia was seen in 12.5% (n=7) patients, which was managed and treated by a Cardiac Electrophysiologist. Of these arrhythmias, ventricular tachycardia, 8.7%,^4^ followed by Paroxysmal AF, 4.2% were most common.^2^ Both VT and AF were managed by administration of IV amiodarone 0.5 mg /kg. There was one case of AVNRT which was managed and reversed by carotid massage. Pulmonary hypertension was seen in 37%(n=17) and heart failure in 8.4% (n=4), and both were managed by a general cardiologist. These cases of heart failure were more associated with hypertension, as seen in a study where hypertensive versus non-hypertensive individuals had heart failure of 70.7% vs. 55.4%, *P* = 0.002, in another study.^20^-^21^

So PPCM is not without its risk and complication but these patients need early booking, timely detection of PPCM by detecting red flag symptoms followed by diagnosis with ECHO, laison with cardiologist and delivery in tertiary hospital. All these factors lead to good outcome and survival. Some clinical parameters like EF percentage, mode of delivery, availability of CCU at back up, BMI, superadded complications of pre-eclampsia can determine cardiac complication and mortality.

### Limitations:

It is a single-center study with a relatively small sample size due to the rarity of peripartum cardiomyopathy and the limited duration of patient recruitment. Therefore, the findings should be interpreted with caution and considered exploratory. Larger multicenter studies are required to validate these observations. Long-term maternal cardiac recovery and future pregnancy outcomes were not evaluated.

### Strengths of study:

Strengths include: Prospective data collection, multidisciplinary management in a tertiary care center and detailed documentation of maternal cardiac complications and fetal outcomes.

## CONCLUSION

Maternal outcomes were generally favorable when patients with peripartum cardiomyopathy were managed at a tertiary care center through a multidisciplinary team approach. Better outcomes were commonly observed in patients with normal BMI, left ventricular ejection fraction above 45.71%, absence of pre-eclampsia and multiple pregnancy.

### Authors Contribution:

**ZAK** review of the article, design and editing of the manuscript.

**FZ:** Manuscript writing, conceived, designed and did statistical analysis & editing of manuscript, is responsible for the integrity of research.

**SJ** reviewed / data collection of all patients.

All authors have approved the final version of the manuscript and are accountable for the integrity of the study.
